# Assessing clinicopathological correlation in chronic traumatic encephalopathy: rationale and methods for the UNITE study

**DOI:** 10.1186/s13195-015-0148-8

**Published:** 2015-10-12

**Authors:** Jesse Mez, Todd M. Solomon, Daniel H. Daneshvar, Lauren Murphy, Patrick T. Kiernan, Philip H. Montenigro, Joshua Kriegel, Bobak Abdolmohammadi, Brian Fry, Katharine J. Babcock, Jason W. Adams, Alexandra P. Bourlas, Zachary Papadopoulos, Lisa McHale, Brent M. Ardaugh, Brett R. Martin, Diane Dixon, Christopher J. Nowinski, Christine Chaisson, Victor E. Alvarez, Yorghos Tripodis, Thor D. Stein, Lee E. Goldstein, Douglas I. Katz, Neil W. Kowall, Robert C. Cantu, Robert A. Stern, Ann C. McKee

**Affiliations:** Alzheimer’s Disease Center, Boston University School of Medicine, 72 East Concord Street, B-7800, Boston, MA 02118 USA; Department of Neurology, Boston University School of Medicine, 72 East Concord Street, Boston, MA 02118 USA; Sports Legacy Institute, 230 Second Avenue, Waltham, MA 02451 USA; Department of Anatomy and Neurobiology, Boston University School of Medicine, 72 East Concord Street, Boston, MA 02118 USA; Data Coordinating Center, Boston University School of Public Health, 715 Albany Street, Boston, MA 02118 USA; VA Boston Healthcare System, U.S. Department of Veterans Affairs, 150 South Huntington Street, Jamaica Plain, MA 02130 USA; Department of Veterans Affairs Medical Center, 200 Springs Road, Bedford, MA 01730 USA; Department of Pathology, Boston University School of Medicine, 72 East Concord Street, Boston, MA 02118 USA; Department of Biostatistics, Boston University School of Public Health, 72 East Concord Street, Boston, MA 02118 USA; Braintree Rehabilitation Hospital, 250 Pond Street, Braintree, MA 02184 USA; Department of Pathology and Laboratory Medicine, Boston University School of Medicine, 72 East Concord Street, Boston, MA 02118 USA; Department of Neurosurgery, Boston University School of Medicine, 72 East Concord Street, Boston, MA 02118 USA; Department of Neurosurgery, Emerson Hospital, 133 Old Road to Nine Acre Corner, Concord, MA 01742 USA

## Abstract

**Introduction:**

Chronic traumatic encephalopathy (CTE) is a progressive neurodegeneration associated with repetitive head impacts. Understanding Neurologic Injury and Traumatic Encephalopathy (UNITE) is a U01 project recently funded by the National Institute of Neurological Disorders and Stroke and the National Institute of Biomedical Imaging and Bioengineering. The goal of the UNITE project is to examine the neuropathology and clinical presentation of brain donors designated as “at risk” for the development of CTE based on prior athletic or military exposure. Here, we present the rationale and methodology for UNITE.

**Methods:**

Over the course of 4 years, we will analyze the brains and spinal cords of 300 deceased subjects who had a history of repetitive head impacts sustained during participation in contact sports at the professional or collegiate level or during military service. Clinical data are collected through medical record review and retrospective structured and unstructured family interviews conducted by a behavioral neurologist or neuropsychologist. Blinded to the clinical data, a neuropathologist conducts a comprehensive assessment for neurodegenerative disease, including CTE, using published criteria. At a clinicopathological conference, a panel of physicians and neuropsychologists, blinded to the neuropathological data, reaches a clinical consensus diagnosis using published criteria, including proposed clinical research criteria for CTE.

**Results:**

We will investigate the validity of these clinical criteria and sources of error by using recently validated neuropathological criteria as a gold standard for CTE diagnosis. We also will use statistical modeling to identify diagnostic features that best predict CTE pathology.

**Conclusions:**

The UNITE study is a novel and methodologically rigorous means of assessing clinicopathological correlation in CTE. Our findings will be critical for developing future iterations of CTE clinical diagnostic criteria.

## Introduction

Chronic traumatic encephalopathy (CTE) is a progressive neurodegenerative disease associated with repetitive head impacts (RHI) [1–6]. Currently, CTE can be diagnosed only pathologically, although clinical research criteria have been proposed [5, 7, 8]. The symptoms of CTE, originally known as “punch drunk” or “dementia pugilistica,” were first described in 1928 in boxers [9]. Pathological evidence of CTE has been observed only in individuals with a history of head impacts that usually were repetitive. Examples of these impacts include those sustained during participation in contact sports, such as American football, soccer, rugby, ice hockey, and professional wrestling [2, 4, 10–14]; blast injuries sustained by military service members [3, 12, 15]; poorly controlled epilepsy; head-banging behaviors; and physical abuse [3, 16, 17].

The pathology of CTE is distinctive and clearly differentiated from other neurodegenerative diseases, such as Alzheimer’s disease (AD) and frontotemporal lobar degeneration (FTLD) [10–12, 17–23]. Gross features of CTE pathology include atrophy of the cerebral cortex (especially the frontal and temporal lobes), diencephalon and mammillary bodies, and cavum septum pellucidum or septal fenestrations [2, 3, 12, 18].

Microscopically, CTE is characterized by the deposition of hyperphosphorylated tau (p-tau) as neurofibrillary tangles, astrocytic inclusions, and neurites irregularly distributed around small blood vessels, preferentially at the depths of cerebral sulci [1–3, 24, 25]. In well-established disease, the tau pathology is most prominent in the frontal and temporal lobes, hippocampus, amygdala, and entorhinal cortex [12, 19, 20].

Symptom onset in CTE usually does not immediately follow RHI, although the length of delay varies widely [2, 3, 12, 19]. A constellation of cognitive, mood, and behavioral symptoms, including impulsivity, aggression, depression, apathy, suicidal ideation, episodic memory loss, and executive dysfunction, have been described [1–5, 12, 26]. Some individuals develop dementia as the disease progresses in severity. Motor symptoms, including gait instability, bradykinesia, and rigidity, are also common, usually late in the disease course [1, 3–5, 12, 26].

To date, understanding of the clinical presentation of CTE has come largely from clinicopathological case studies of individuals exposed to RHI based on clinical interviews with family members after the individuals’ death [4, 26]. Owing to the recall bias inherent to retrospective studies and the ascertainment bias associated with brain donation, there is a clear need for long-term, prospective, longitudinal studies. However, those studies will require a time period of nearly a decade or more before sufficient data can be generated to draw conclusions. As those longitudinal studies are developed, it is important to establish and refine consensus criteria for clinical and pathological CTE diagnosis and to continue gathering retrospective data to inform the long-term prospective studies. The imperative to continue analyzing clinical course and pathological correlates of CTE using cost-effective, efficient, and immediately feasible retrospective study designs is especially critical, given the potential long-term health risks of RHI.

Understanding Neurologic Injury and Traumatic Encephalopathy (UNITE) is a recently funded National Institutes of Health (NIH) U01 project in which researchers are examining the neuropathology and clinical symptoms of brain donors who have experienced RHI. The aim of the project is to study the relationship between clinical symptoms and the neuropathology of CTE.

Here, we present the methodology of the UNITE study for examining clinicopathological correlation. Specifically, we set forth the following aims: (1) to investigate the validity of recently proposed clinical criteria for CTE [5] using a clinical consensus meeting and recently validated neuropathological criteria for CTE ([2, 27], (A.C. McKee) as a gold standard for CTE diagnosis; (2) to identify sources of error between clinical consensus diagnosis and the neuropathological diagnosis of CTE; and (3) to identify individual diagnostic features that collectively best predict CTE pathology.

## Methods

**Fig. 1:**
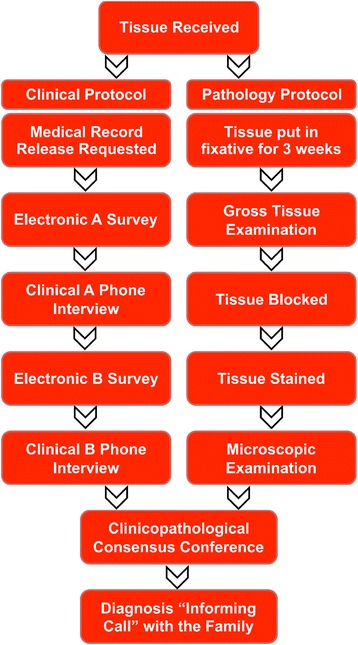
Overall flowchart of the study methodology

The institutional review board at the Boston University (BU) Medical Campus approved all of our research activities (IRB number 31614). Because all participants are deceased, consent does not need to be obtained. Figure 1 shows an overall flowchart of the study methodology. The presented methodology resulted from several iterations of updates based on investigator feedback.

### Study recruitment

**Fig. 2:**
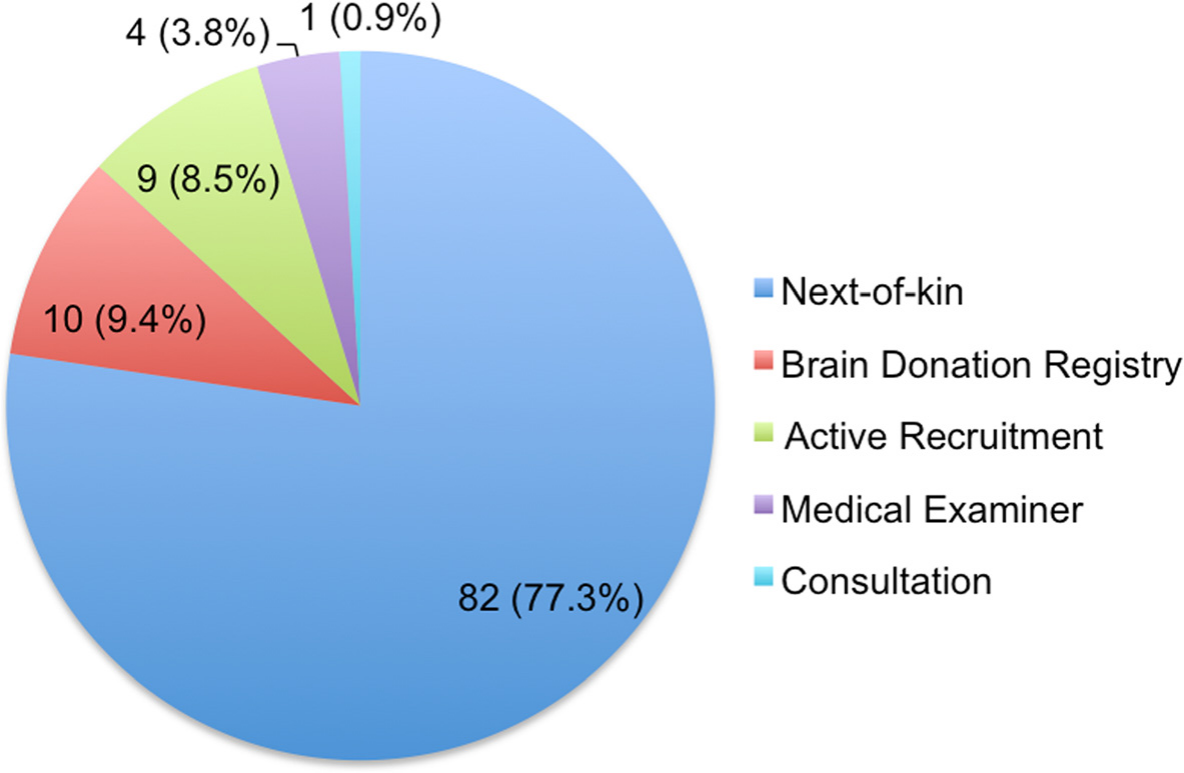
Recruitment mechanisms in place at the U.S. Department of Veterans Affairs–Boston University–Concussion Legacy Foundation Brain Donation Registry and Brain Bank since Understanding Neurologic Injury and Traumatic Encephalopathy project recruitment began. Next-of-kin recruitment: A potential donor’s legal next of kin contacts the brain bank near the time of death to ask about participation. Active recruitment: A member of the brain bank staff contacts a potential donor’s next of kin near the time of death to ask about participation. Brain Donation Registry: A potential donor contacts the brain bank and pledges to donate upon death. Medical examiner: A medical examiner contacts the brain bank upon suspicion of a diagnosis of chronic traumatic encephalopathy or if an individual’s family member expresses to the medical examiner interest in brain donation. Consultations: A neuropathologist contacts the brain bank to release tissue for further evaluation

The U.S. Department of Veterans Affairs (VA)-BU-Concussion Legacy Foundation (CLF) Brain Donation Registry and Brain Bank is a collaborative effort of the CTE Research Program within the BU Alzheimer’s Disease Center (ADC), the VA Boston Healthcare System, and CLF, a non-profit organization dedicated to brain trauma research and education. Subject recruitment is ongoing and will occur throughout the 4-year study period. Figure 2 shows recruitment mechanisms in place since UNITE recruitment began in January 2014. For the majority of the brain donors, the subjects’ next of kin contact the brain bank and agree to donate near the time of death. While living, some study subjects agree, through the Brain Donation Registry, to donate their brain and spinal cord after death. Potential subjects can register at any time, provided they meet specific inclusion and exclusion criteria (detailed below). The registry currently has nearly 600 potential living subjects. We anticipate that several hundred more will join the registry over the 4-year study period. On the basis of the rate of brain donation in recent previous work, we anticipate that 300 subject specimens will be donated over the 4-year study period, with 20 % acquired through the registry.

### Eligibility criteria

Regardless of recruitment mechanism, potential subjects are evaluated using the same inclusion and exclusion criteria (Table 1). The inclusion criteria are based solely on RHI exposure history, regardless of whether symptoms are present. The criteria allow for breadth of RHI exposure (e.g., athletics, military service, abuse) while requiring sufficient intensity such that there is a reasonable chance for the development of CTE (based on our experience, acknowledging that the relationship between RHI and CTE is still under investigation). The inclusion criteria are broader for women, who historically have been investigated less thoroughly than men, and for individuals with amyotrophic lateral sclerosis (ALS), a particular research focus at our center. The exclusion criteria prevent inclusion of brain and spinal cord specimens of poor quality.

**Table 1:** Inclusion and exclusion criteria for the UNITE study

Descriptions	
Inclusion criteria	
1.	Men who played American football or ice hockey at the professional or Olympic level or who played at the collegiate, semiprofessional, or junior level for at least 2 years
2.	Men who played a high-risk contact sport, other than American football or ice hockey, at the professional or Olympic level
3.	Women who played a high-risk contact sport at the professional, Olympic, or collegiate level
4.	Men or women who played a high-risk contact sport at the professional, Olympic, collegiate, or high school level who died before the age of 35 years
5.	Men or women who played a high-risk contact sport at the professional, Olympic, collegiate, or high school level who were diagnosed with ALS during life
6.	Men or women with a military history of combat exposure
7.	Women with a history of domestic abuse
Exclusion criteria	
1.	Specimen with a postmortem interval before fixation of longer than 72 h
2.	Specimen that was embalmed following brain autopsy
3.	Specimen of less than one full hemisphere

ALS, Amyotrophic lateral sclerosis; UNITE, Understanding Neurologic Injury and Traumatic Encephalopathy

### Consent

Consent for donation of brains and spinal cords is acquired from the decedent’s legal next of kin or legally authorized representative (LAR). The next of kin or LAR also may consent for the donation of cerebrospinal fluid (CSF), blood, and/or eye tissue for use in related studies.

### Brain acquisition

A member of the research team coordinates the extraction and shipment of the specimen. A properly trained individual (pathologist, medical examiner, autopsy technician, diener) extracts the tissue locally. If immediate shipment is possible, the specimen is placed on wet ice and shipped using a courier service to minimize the postmortem interval. If immediate shipment is not possible, the specimen is placed in 10 % formalin for fixation for a minimum of 2 weeks before shipment.

### Pathological processing and evaluation

The detailed methodology used for pathological processing and evaluation has been published previously [28, 29]. The McKee Laboratory evaluates brains obtained from several brain banks, including -Concussion Legacy Foundation, the Boston University-Alzheimer's Disease Center, the Framingham Heart Study, the New England Centenarian Study, the National Registry of Veterans with Amyotrophic Lateral Sclerosis, and the Veterans Administration National Posttraumatic Stress Disorder Brain Banks. Regardless of individuals’ brain bank membership, all brains are processed identically and assigned a random identification number that does not identify the brain bank to which they belong. Briefly, for fresh tissue, quality control measures are followed, including RNA integrity number (Agilent Technologies, Santa Clara, CA, USA) and pH. The brain is hemisected, then one half is sectioned and frozen and the other half is fixed for 3 weeks. The fixed tissue is dissected and processed into tissue sections, including paraffin-embedded tissue sections and large, fixed coronal slabs. Tissue blocks and stains are detailed in Table 2. If screening regions are positive for β-amyloid, α-synuclein, or phosphorylated transactive response DNA binding protein 43 kDa (pTDP-43), additional regions are stained to allow for complete staging of these pathologies. Positive and negative controls are stained simultaneously to identify improperly stained material.

**Table 2:** Brain tissue blocks and stains used for neuropathology

		Stain							
		Reserve	Luxol fast blue	Bielschowsky silver stain	AT8	Amyloid-β (4G8)^a^	α-Synuclein^a^	pTDP-43^a^	Minimum staining^b^
Brain region	Paraffin blocks, n								
Olfactory bulb	1		X		X		X		X
Midbrain at level of red nucleus	1		X		X	X	X	X	
Midbrain at superior cerebellar peduncle	1	X							
Precentral, postcentral cortices (BA 4, 3, 2, 1)	1		X		X				
Inferior parietal cortex (BA 39, 40)	1		X	X	X	X			X
Anterior cingulate (BA 24)	1		X				X		
Superior frontal (BA 8, 9)	1		X		X				X
Inferior frontal cortex (BA 10, 11, 12)	2		X		X				X
Dorsolateral frontal (BA 45, 46)	2		X	X	X	X		X	X
Caudate-putamen-accumbens, septal cortex	2		X		X	X			
Insular cortex	2		X		X				
Temporal pole (BA 38)	1		X		X				X
Superior temporal (BA 20, 21, 22)	1		X		X	X			X
Amygdala, with entorhinal cortex (BA 28)	1		X		X	X	X	X	X
Globus pallidus, insula, substantia innominata	1		X		X				
Anterior hippocampus	1	X							
Hippocampal formation, lateral geniculate	1		X	X	X	X		X	X
Superior temporal posterior (BA 41, 42)	1	X							
Thalamus with subthalamic nucleus	1	X							
Hypothalamus, mammillary body	1		X		X				
Posterior thalamus	1	X							
Posterior cingulate (BA 23, 31)	1	X							
Calcarine cortex (BA 17, 18)	1		X	X	X				
Superior parietal cortex (BA 7B)	1	X							
Upper pons (level of locus coeruleus)	1		X		X				X
Pons, middle cerebellar peduncle	1	X							
Medulla oblongata with inferior olives	1		X		X		X		X
Cervical spinal cord	1		X		X			X	
Thoracic spinal cord	1	X							
Lumbar spinal cord	1		X						
Sacral spinal cord	1	X							
Cerebellar vermis	1		X		X				
Cerebellum with dentate nucleus	1		X	X	X	X			X
Parastriate cortex	1	X							

*Abbreviations: BA* Brodmann area, *pTDP-43* phosphorylated transactive response DNA binding protein 43 kDa

^a^If screening regions are positive for β-amyloid, α-synuclein, or pTDP-43, additional regions are stained to allow for complete staging of these pathologies

^b^Minimum regions that must be evaluated to make a neuropathological chronic traumatic encephalopathy diagnosis

The pathological evaluation and diagnosis occur without any knowledge of the subject’s RHI or clinical history and are confirmed by two neuropathologists (ACM, TDS). Semiquantitative measures of phosphorylated tau burden (by AT8 immunostaining), β-amyloid deposition (by 4G8 immunostaining for Thal phase and Bielschowsky silver stain for Consortium to Establish a Registry for Alzheimer’s Disease score), α-synuclein–positive Lewy body and neurite burden, pTDP-43 burden, vascular disease, and neuronal loss are recorded for prespecified regions. The validated criteria for diagnosis ([2, 27], A.C. McKee) and stages [2] of CTE are summarized in Tables 3 and 4. Well-established pathological criteria are used for diagnosis of all comorbid diseases, including ALS [30, 31], AD [32–37], Parkinson’s disease and Lewy body disease [38–40], FTLD (including progressive supranuclear palsy, corticobasal degeneration, and Pick’s disease) [41–45], and multiple system atrophy [43]. A neuropathology report is generated that includes a description of the macroscopic and microscopic findings and a list of pathological diagnoses, including CTE stage.

**Table 3:** Pathological criteria used for CTE diagnosis

Descriptions	
Defining criteria	
1.	Perivascular accumulation of abnormal hyperphosphorylated tau within neurons, astrocytes, and/or cell processes in the neocortex
2.	Irregular distribution of p-tau–immunoreactive cells and processes at the depths of cerebral sulci
Supportive criteria	
3.	Macroscopic abnormalities in the septum pellucidum (cavum, fenestration), disproportionate dilation of the third ventricle or signs of previous brain injury
4.	Abnormal tau-immunoreactive neuronal lesions affecting the neocortex predominantly in superficial layers 2 and 3, as opposed to layers 3 and 5 as in AD

*Abbreviations: AD* Alzheimer’s disease, *CTE* chronic traumatic encephalopathy

These criteria are based on previous publications [2, 27] and A.C. McKee

**Table 4:** CTE pathological stages

Stages	Descriptions
1	Discrete perivascular p-tau foci in the neocortex, usually found at the depths of sulci
2	Multiple perivascular p-tau foci in the neocortex, typically at the depths of the cerebral sulci, with involvement of superficial layers of adjacent cortex and sparing of the medial temporal lobe structures
3	Widespread p-tau lesions in the frontal, temporal, parietal, insular, and septal neocortices, most severe at the depths of the sulci and in the superficial cortical layers, with involvement of the entorhinal cortex, amygdala, and hippocampus
4	Diffuse pathology throughout the cerebral cortex and the medial temporal structures, sparing the calcarine cortex

*Abbreviation: CTE* chronic traumatic encephalopathy, *p-tau* phosphorylated tau

CTE pathological stages are based on extent and anatomic distribution of p-tau pathology [2]

### Retrospective clinical evaluation

The goal of the retrospective clinical evaluation is to obtain each subject’s demographic information; RHI exposure; substance use; and medical, social, and family histories, with a particular focus on possible neurodegenerative conditions, including symptom breadth, severity, and progression. The retrospective clinical evaluation comprises a combination of online surveys and telephone calls between researchers and the family members and close friends of the subject. Data are collected through an unstructured interview with either a behavioral neurologist or a neuropsychologist and with modified (for completion retrospectively, following death, by informants) versions of standardized, validated scales (Table 5). Preference was given to scales already in use in other relevant BU studies, including studies of CTE and AD [46–49], and to scales that are included in the National Institute of Neurological Disorders and Stroke (NINDS) Common Data Elements. Researchers conducting these evaluations are completely blinded to the pathological examinations and findings.

**Table 5:** Administered clinical scales

Scales	Clinical evaluation sections
Ohio State University TBI Identification Method Short Form [50]	Clinical Interview A
Geriatric Depression Scale [51]	Online Survey B
Cognitive Difficulties Scale [52]	
Behavioral Rating Inventory of Executive Function–Adult Version [53]	
BIS-11 [54]	
Apathy Evaluation Scale [55]	
ALS Functional Rating Scale^a^ [56]	
Functional Assessment Questionnaire [57]	Clinical Interview B
Clinician Assessment of Fluctuations [58]	
Brown-Goodwin Aggression Scale [59]	
Structured Clinical Interview for DSM-IV [60]	

*Abbreviations: ALS* Amyotrophic lateral sclerosis; *DSM-IV* Diagnostic and Statistical Manual of Mental Disorders, Fourth Edition, *TBI* Traumatic brain injury

All scales were adapted to account for data being collected retrospectively from an informant

^a^Administered only for subjects diagnosed with ALS during life

There are five parts to the clinical evaluation: two online surveys (termed Online Surveys A and B), two telephone interviews (termed Clinical Interviews A and B), and a medical record review. Informants may complete the online surveys individually or as a group (i.e., several members of the decedent’s family responding together). For the clinical interviews, informants participate as a group. A behavioral neurologist or neuropsychologist (termed the lead clinician) conducts Clinical Interview A, and a research assistant conducts Clinical Interview B. To assess informant reliability, informants answer questions pertaining to the nature and duration of their relationship with the subject and the frequency with which they were in contact with the subject. A description of each part of the clinical evaluation (presented in the order in which the data are collected) is provided in the subsections below.

#### Online Survey A

Online Survey A queries the subject’s demographic information, educational attainment, occupational history, living situation before death, athletic history (type of sports played, level, position, age of first exposure, and duration), and military history (branch, location of service, and duration of combat exposure). The survey uses a nested question structure with skip logic to ensure that questions are appropriately tailored to each subject.

#### Clinical Interview A

During Clinical Interview A, the clinician (a behavioral neurologist or a neuropsychologist) obtains a detailed medical history, including traumatic brain injuries (TBIs), and recreates a timeline of cognitive, behavioral, and mood symptomatology. Specifically, the clinician asks semistructured questions about cause of death, medical history (including vascular risk factors), neurological history (including risk factors for cognitive and motor impairment), and psychiatric history. The clinician then asks semistructured questions about mild to severe TBIs using the Ohio State University TBI Identification Method Short Form [50] and two questionnaires, adapted from published studies, that address military-related head injuries and concussions that result in even the mildest symptoms [47, 48]. Finally, using unstructured questions, the clinician obtains a precise chronology of deficits in cognition (memory, executive function, attention/concentration, language, visuospatial function), behavior and/or mood (depression, apathy, mania, anxiety, irritability/anger, abusiveness, social inappropriateness, psychosis), and daily function (including instrumental activities of daily living). Motor functioning, sleep, headaches, substance use, and family history are queried in the same manner. Once the interview is completed, the clinician answers several summary questions about predominant symptoms (cognitive, mood, behavior, motor), symptom onset, and disease progression.

#### Online Survey B

Online Survey B is used to collect data about the subject’s cognition, mood, and behavior (including impulsivity and apathy) through the administration of the following validated scales: Geriatric Depression Scale Short Form [51], Cognitive Difficulties Scale (CDS) [52], Behavior Rating Inventory of Executive Function–Adult Version [53], Barratt Impulsivity Scale version 11 (BIS-11) [54], and Apathy Evaluation Scale (AES) [55]. For subjects diagnosed during life with ALS, the ALS Functional Rating Scale [56] is also administered.

#### Clinical Interview B

In Clinical Interview B, the researcher asks informants semistructured questions to quantify information obtained qualitatively in Clinical Interview A. Some questions from Online Survey B are repeated for quality control. Specifically, family history is obtained using modified questions from the National Alzheimer’s Coordinating Center Uniform Data Set [49]. Cognitive functioning, including memory, language, attention, executive function, and visuospatial function, is assessed using selected questions from the informant section of the CDS [52]. Daily function, cognitive fluctuations, and aggression are assessed using the Functional Assessment Questionnaire [57], the Clinician Assessment of Fluctuations [58], and the Brown-Goodwin Aggression Scale [59], respectively. Impulsivity and apathy are assessed using selected questions from the BIS-11 [54] and the AES [55], respectively. The presence of major depressive disorder, bipolar disorder, anxiety disorders (including panic disorder, obsessive compulsive disorder, agoraphobia, social anxiety disorder, specific phobias, generalized anxiety disorder, and posttraumatic stress disorder), psychosis, substance use, and somatoform disorders is assessed using modified questions from the Structured Clinical Interview for DSM-IV [60]. Sleep and headache are briefly assessed using modified questions from the Mayo Sleep Questionnaire [61] and the Cleveland Clinic Headache Intake Questionnaire, respectively [62]. Motor function is assessed using questions developed internally to query symptoms of parkinsonism.

#### Medical record review

For all incoming cases, we request permission from the subject’s legal next of kin or LAR to review medical records as well as the names and locations of health care providers. A research assistant contacts the health care providers to request the records, including original brain imaging (rather than just a report). The research assistant initially reviews all records and extracts salient information, including psychiatric, neurological, and neuropsychological evaluations; brain imaging; medical history; and medications. A behavioral neurologist and a neuropsychologist then review the extracted information, including original images if available. Information gathered during the medical record review is combined with the data gathered in the previous steps to complete the clinical evaluation.

### Clinicopathological consensus conference

**Fig. 3:**
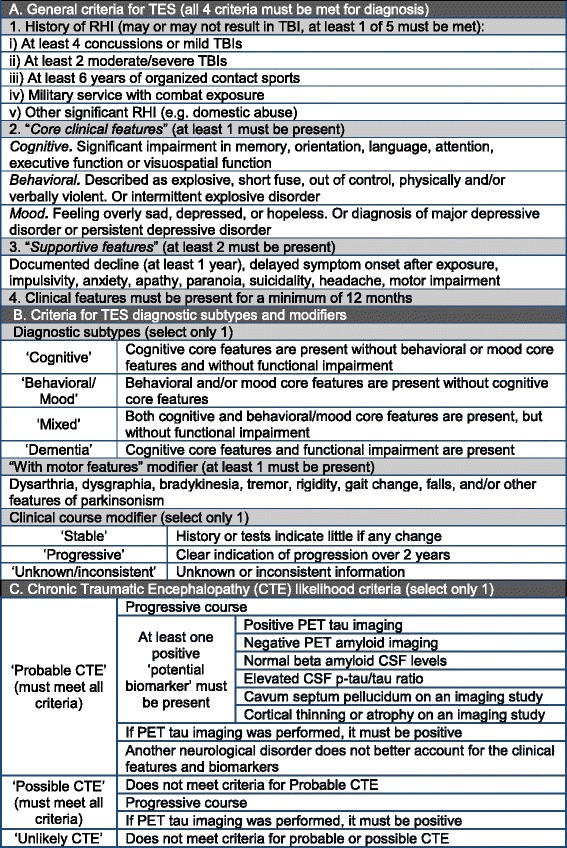
Adapted clinical diagnostic criteria for traumatic encephalopathy syndrome^a^. Abbreviations: CSF cerebrospinal fluid, CTE chronic traumatic encephalopathy, PET positron emission tomography, p-tau phosphorylated tau, RHI repetitive head injuries, TBI traumatic brain injury, TES traumatic encephalopathy syndrome. ^a^Criteria adapted from Montenigro et al. [5]

Clinical consensus methodology is based on recommendations of Bertens et al. [63]. At a twice-monthly clinicopathological consensus conference (CPC), a panel of doctoral-level clinicians reaches a clinical consensus diagnosis using clinical research criteria [38, 61, 64–69], including those recently proposed for CTE [5] (Fig. 3). The clinical panel is composed of neuropsychologists, neurologists, psychiatrists, and neurosurgeons who specialize in neurodegenerative disease and/or TBI. At least three and upward of six panel members are present for each CPC.

For each case, a lead clinician reads a standardized clinical summary based on the information collected during the retrospective clinical evaluation. This summary outlines the disease course. It also includes age at death and cause of death; a subjective assessment of informant reliability; prior athletic, military, and TBI history; past medical, educational, and occupational history; living situation before death; substance use history; and family history. Last, it includes salient features in medical records, including neuropsychological testing, neuroimaging (including a reading from a behavioral neurologist if original images are available), CSF biomarkers, diagnoses made during life, and medications prescribed. At the conclusion of the clinical presentation and before any formal discussion, each clinical consensus member votes independently, without discussion, on whether criteria for traumatic encephalopathy syndrome (TES) [5] are met. The diagnosis of TES, which incorporates CTE, is made on the basis of criteria outlined by Montenigro et al. [5], with modification because neuropsychological testing may not have been conducted during the subject’s life. Although several groups have proposed CTE clinical criteria [7, 8], use of the Montenigro et al. criteria provides several advantages. To be included in the core criteria, signs and symptoms needed to be frequent (70 %) among cases diagnosed neuropathologically with CTE using the criteria proposed by McKee et al. [2] and free of comorbid neurodegenerative disease. The criteria also are operationalized for research, explicitly defining the minimum required exposure level, supportive features, subtype designations, potential biomarkers, and relative likelihood of CTE (probable, possible, or unlikely). Of note, TES is a broad umbrella term meant to describe the clinical presentation of CTE as well as other possible long-term consequences of RHI, including other neurodegenerative diseases. A TES diagnosis neither necessitates a possible or probable CTE diagnosis nor excludes another clinical neurodegenerative diagnosis [5].

**Fig. 4:**
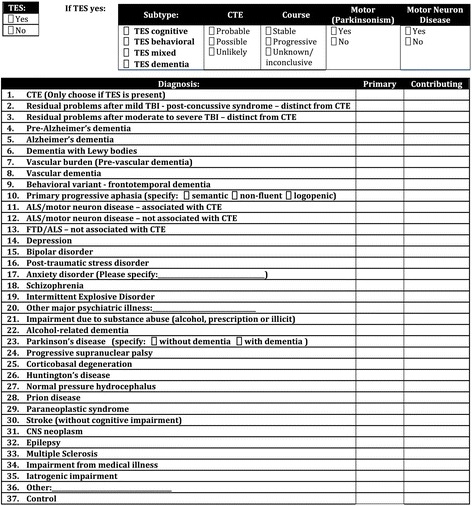
Clinical consensus diagnostic form completed by each clinician. *ALS* amyotrophic lateral sclerosis, *CNS* central nervous system, *CTE* chronic traumatic encephalopathy, *FTD* frontotemporal degeneration, *TBI* traumatic brain injury, *TES* traumatic encephalopathy syndrome

If the criteria for TES are met, the clinician indicates the subtype designations and the relative likelihood of underlying CTE (probable, possible, or unlikely) based upon additional supportive features [5]. The clinicians also record a primary clinical diagnosis and, if appropriate, contributing clinical diagnoses. Figure 4 shows the diagnostic form that each clinician completes. After each clinician, blinded to the other clinicians’ diagnoses, completes and submits the diagnostic form, the group discusses the case. The discussion includes questioning of the lead clinician about specific details with the goal of reaching a consensus diagnosis using a format identical to that used for the previous independent voting. To reach consensus, a majority of the clinicians present must agree on the diagnosis. Once a consensus diagnosis is reached, panel members again complete a written diagnostic form as a means to record dissent from the consensus.

After the clinicians reach a consensus, the neuropathologist who evaluated the case presents the pathological findings. The presentation includes the brain weight, gross and microscopic images, and an overall summary. The presentation focuses on 1) regional patterns of cerebral and white matter atrophy, 2) evidence of septal abnormalities, including cavum septum pellucidum or fenestrations, 3) pallor of the substantia nigra and locus coeruleus, 4) extent and anatomic distribution of neuronal loss and gliosis, 5) immunohistochemistry (p-tau, β-amyloid, pTDP-43 and α-synuclein) and 6) vascular pathology. The summary includes pathological diagnoses with staging when appropriate. All neuropathological diagnoses and associated reports are completed before the consensus conference and without knowledge of the subject’s antemortem exposure history or clinical presentation, and they are not changed on the basis of clinicians’ diagnoses or discussions.

After the clinical and pathological presentations, the clinicians and pathologists discuss clinicopathological correlation. For each case, the physicians and neuropsychologists identify key summary features of the case that help inform future research directions. For cases with discrepancies between the clinical and pathological findings, the cause of the differences and how the discrepancies contribute to diagnostic uncertainty are discussed.

Last, the lead clinician and pathologist present both the clinical and pathological diagnoses to the informants by telephone. The informants are also provided with a written report that summarizes the diagnoses.

### Data analysis

The data analysis will involve three major phases. Phase 1 will evaluate the reliability of the consensus raters and the validity of the clinical research criteria for CTE and the consensus process [63, 70]. For our primary analyses, we will consider a dichotomous clinical diagnosis (i.e., possible or probable CTE vs. no CTE). First, we will calculate the pre- and postconsensus interrater reliability (Cohen’s κ) between clinical consensus members [63, 70, 71], along with the standard errors and 95 % confidence intervals. Next, we will calculate the pre- and postconsensus sensitivity, specificity, and accuracy of the clinical diagnosis using the presence of CTE pathology as a gold standard. In secondary analyses, we will assess the reliability and validity of clinical subtypes and an alternative operationalization of the likelihood of CTE (i.e., probable CTE vs. possible or no CTE).

Phase 2 of the analysis involves an evaluation of sources of diagnostic disagreement between clinical consensus members and sources of diagnostic error between the consensus diagnosis and the pathological diagnosis. For each case with diagnostic disagreement (either pre- or postconsensus), we will review the case and identify the issues leading to the disagreement. We will review how these major issues differ pre- and postconsensus, paying particular attention to those that remain or that occur postconsensus. Similarly, for each case with a consensus diagnostic error, we will review the case and identify the major issue leading to the error. We will calculate the frequency of each type of diagnostic error (e.g., frequency of clinical consensus diagnosis of CTE with pathological AD). Finally, we will review differences in diagnostic errors between false positives and false negatives.

Phase 3 involves the analysis of individual diagnostic features (examples include but are not limited to contact sport position, years and level of play, number and severity of individual TBIs, memory impairment, depression, impulsivity, and motor impairment). We will calculate the sensitivity and specificity of each feature using pathological CTE as a gold standard. Among cases of pathological CTE, we will calculate the frequency of each feature overall, as the presenting symptom and/or sign, as an early symptom and/or sign, and as a late symptom and/or sign. Next, we will create statistical models of pathological CTE risk, using these diagnostic features as predictors. We will use conventional logistic regression as well as two machine learning algorithms: random forests and elastic net penalized logistic regression. The machine learning algorithms account for the often erroneous assumptions of classical logistic regression that predictors are not correlated with each other and that their effects are additive [72]. Diagnostic features with the best sensitivity and specificity, and that best predict CTE pathology, will be critical to include in future iterations of clinical diagnostic criteria for CTE.

## Discussion

The last decade has seen an increased interest in understanding the relationship between RHI and the development of neurodegenerative disorders, most centrally CTE. As CTE is closely linked to participation in contact sports such as American football and to head injuries sustained by soldiers participating in the conflicts in Iraq and Afghanistan, the topic also has garnered considerable attention from the media and the public at large. Recently, the NIH, the National Football League (NFL), and the U.S. Department of Defense sponsored further research to elucidate the connection between RHI and the development of CTE [73–75]. The UNITE study is part of this initiative. Here, we describe the UNITE study methodology for examining clinicopathological correlation in CTE.

The UNITE methodology has several strengths, including extensive and carefully designed data collection and the use of best practices to reach a clinical consensus diagnosis [63]. Retrospective clinical data collection from informants includes an unstructured interview, a structured interview, two online surveys, and a review of medical records. In the unstructured interview, an experienced behavioral neurologist or neuropsychologist obtains a comprehensive disease timeline, similarly to an initial clinical visit, so that the case can be presented in detail at a consensus conference. Via the structured interview and online surveys, data are collected that can be coded and analyzed to answer questions about signs and symptoms of disease, independently of the consensus diagnosis. Pathological data collection includes exhaustive sampling of brain regions and comprehensive immunohistochemical analysis. All vascular and neurodegenerative pathological diagnoses are made on the basis of recently validated CTE criteria ([2, 27], A.C. McKee) or other well-established criteria.

In designing our methodology for reaching a clinical consensus diagnosis, we followed best practices, paying careful attention to the evaluating panel’s constitution, information presented to the panel, and the diagnostic decision-making process for consensus [63, 76]. Our panelists have diverse training (behavioral neurology, neurosurgery, neuropsychology, brain injury and rehabilitation, and psychiatry) and decades of professional experience in areas germane to the study of CTE, including neurodegenerative disease and TBI. The information presented to the panel is based on methodology used in the study of AD [77] and consists of a written summary of the disease course and medical, social, and family histories [1, 5, 7, 26]. The diagnostic decision-making process follows recommended procedures. Consensus members make an individual preliminary diagnosis before discussion with other panel members. Consensus diagnosis, subtype designations, and likelihood of CTE are determined by majority vote after a panel discussion. Individual dissension from the consensus diagnosis is recorded for later analysis [63].

Our study design allows not only for testing the validity of clinical research criteria and the consensus process but also for recognizing sources of error and identifying diagnostic features that best predict CTE pathology. We have proposed several approaches in the Data Analysis section above to conduct these analyses that will allow us to move past the traditional case series approach that has largely defined CTE research to date. For example, whereas there are clear pathological differences between CTE and AD, understanding and differentiating the clinical symptoms, particularly in older adults, remains a significant challenge [78]. Analyzing the cases collectively using a quantitative approach will help us better understand the clinical distinctions between these diseases. Clinical diagnostic features that best predict CTE pathology will be critical to include in future iterations of clinical diagnostic criteria for TES and CTE. In particular, as potential in vivo biomarkers for CTE are developed, the UNITE methodology will provide a critical mechanism by which to examine their predictive validity.

We strive to be methodologically rigorous, but several potential limitations need to be highlighted. In any study involving brain donation, there is clear ascertainment bias. Even though brain donors are selected on the basis of their exposure to repetitive brain trauma, families that donate are more likely to have witnessed symptoms during the donor’s life [26]. Ascertainment bias increases the probability of CTE being present at autopsy and may also limit variability and increase the mean severity of clinical presentation. All of these factors in turn could affect the reliability and validity of the consensus diagnosis. To address this bias, our recruitment network actively encourages the recruitment of subjects without or with limited symptoms who were exposed to RHI. As more asymptomatic brain donors are included in the study, the generalizability of our findings will increase.

Because we ask informants to recall information from years earlier, there is potential for recall bias. Informants may recall events or symptoms more clearly if they occurred closer to the time of death or if they strongly affected the informant. Further, if the informants did not witness events, they may be unaware or poorly informed of what occurred. This is especially common for subjects’ children who may not have been living or who were very young when RHI occurred. We have tried to reduce recall bias by making our structured interview comprehensive so that informants must only recognize that symptoms were present rather than need to freely recall that they occurred. We also pay special attention to the age at individual symptom onset.

Because we do not evaluate subjects during their lives, the lead clinician cannot present firsthand observations of the subject and standardized, objective data, including neuropsychological and biomarker data, cannot be collected. The lack of these data introduces error into the clinical diagnoses. Although we carefully review medical records, including clinicians’ impressions and neuropsychological, imaging, and CSF data, the medical record data we collect are neither universal nor uniform. Further, there may be discrepancies between the data collected from informants and medical record data. Because the structured data collection is standardized and these data are present for all subjects, we plan to use these data alone for our item-level analyses. However, for the consensus diagnosis, we consider all data—structured, unstructured, and from the medical record review. It is up to the lead clinician to present a cogent history to the consensus group, acknowledging to the group any discrepancies in the data.

Clinicopathological studies are not designed to assess causality. To assess whether trauma is a cause of CTE in a human study would require precise longitudinal monitoring of brain trauma exposure and a method to detect and monitor the presence of CTE during life, neither of which is currently possible. Numerous case reports and case studies have strongly suggested a direct association between RHI and the development CTE [2, 10–12, 15, 26, 79], and all cases of pathologically confirmed CTE have included a history of RHI. The goal of the UNITE study is not to establish this causal link, but rather to examine clinicopathological correlation. However, given the strong association between RHI and CTE, RHI is a central component of the UNITE inclusion criteria.

Finally, the retrospective clinicopathological approach has been used successfully to characterize neurodegenerative disease over the past century [74, 75]. In AD, the development of clinical diagnostic criteria [64] and the retrospective validation of these criteria using consensus diagnosis and neuropathology as a gold standard [77] long preceded prospective longitudinal validation. Similar retrospective methodology also was used for other neurodegenerative diseases, including the behavioral variant of frontotemporal dementia [61].

## Conclusions

In the 20 months of active study recruitment to date, our team has successfully brought 99 cases to consensus and an additional 28 brain donations are awaiting consensus evaluation. When combined with previous cases, the VA-BU-CLF brain bank currently holds 172 pathologically confirmed cases of CTE, representing the vast majority of cases of CTE reported to date [80]. This collection of CTE cases has already led to important advances in understanding of the pathobiology of CTE [2, 12, 21, 81] and has been instrumental in the initial stages of validation of the neuropathological criteria for CTE [2]. Although beyond the scope of this article, UNITE has several additional goals, including imaging of ex vivo tissue to guide the development of CTE biomarkers, investigating neurodegenerative comorbidity in CTE, staging of CTE disease severity, modeling RHI as risk factor for CTE, and evaluating genes and gene–environment interactions as a risk factor for CTE. In coordination with NIH biobanks and the Federal Interagency Traumatic Brain Injury Research informatics system, the VA-BU-CLF brain bank and the UNITE study will be used to establish a multisite biospecimen and data repository that will make tissue and data available to qualified investigators around the world who are studying the effects of RHI. These initiatives funded by NINDS and the National Institute of Biomedical Imaging and Bioengineering also will be fundamental to understanding CTE pathogenesis, determining therapeutic research targets, and advancing the clinical diagnosis of CTE.

## Abbreviations

AD: Alzheimer’s disease
ADC: Alzheimer’s Disease Center
AES: Apathy Evaluation Scale
ALS: Amyotrophic lateral sclerosis
BA: Brodmann area
BIS-11: Barratt Impulsivity Scale version 11
BU: Boston University
CDS: Cognitive Difficulties Scale
CLF: Concussion Legacy Foundation
CPC: Clinicopathological consensus conference
CSF: Cerebrospinal fluid
CTE: Chronic traumatic encephalopathy
FTLD: Frontotemporal lobar degeneration
LAR: Legally authorized representative
NFL: National Football League
NIH: National Institutes of Health
NINDS: National Institute of Neurological Disorders and Stroke
PET: Positron emission tomography
p-tau: Hyperphosphorylated tau
pTDP-43: Phosphorylated transactive response DNA binding protein 43 kDa
RHI: Repetitive head impacts
TBI: Traumatic brain injury
TES: Traumatic encephalopathy syndrome
UNITE: Understanding Neurologic Injury and Traumatic Encephalopathy
VA: U.S. Department of Veterans Affairs
